# Supplementary material for: Structural basis of the interaction between SETD2 methyltransferase and hnRNP L paralogs for governing co-transcriptional splicing
Source: Nat Commun. 2021 Nov 8;12:6452. doi: 10.1038/s41467-021-26799-3 (PMC8575775; doi:10.1038/s41467-021-26799-3)
Supplement: Supplementary file 1 — Supplementary Information [file 41467_2021_26799_MOESM1_ESM.pdf]

Structural basis of the interaction between SETD2 methyltransferase and hnRNP L paralogs for governing co-transcriptional splicing

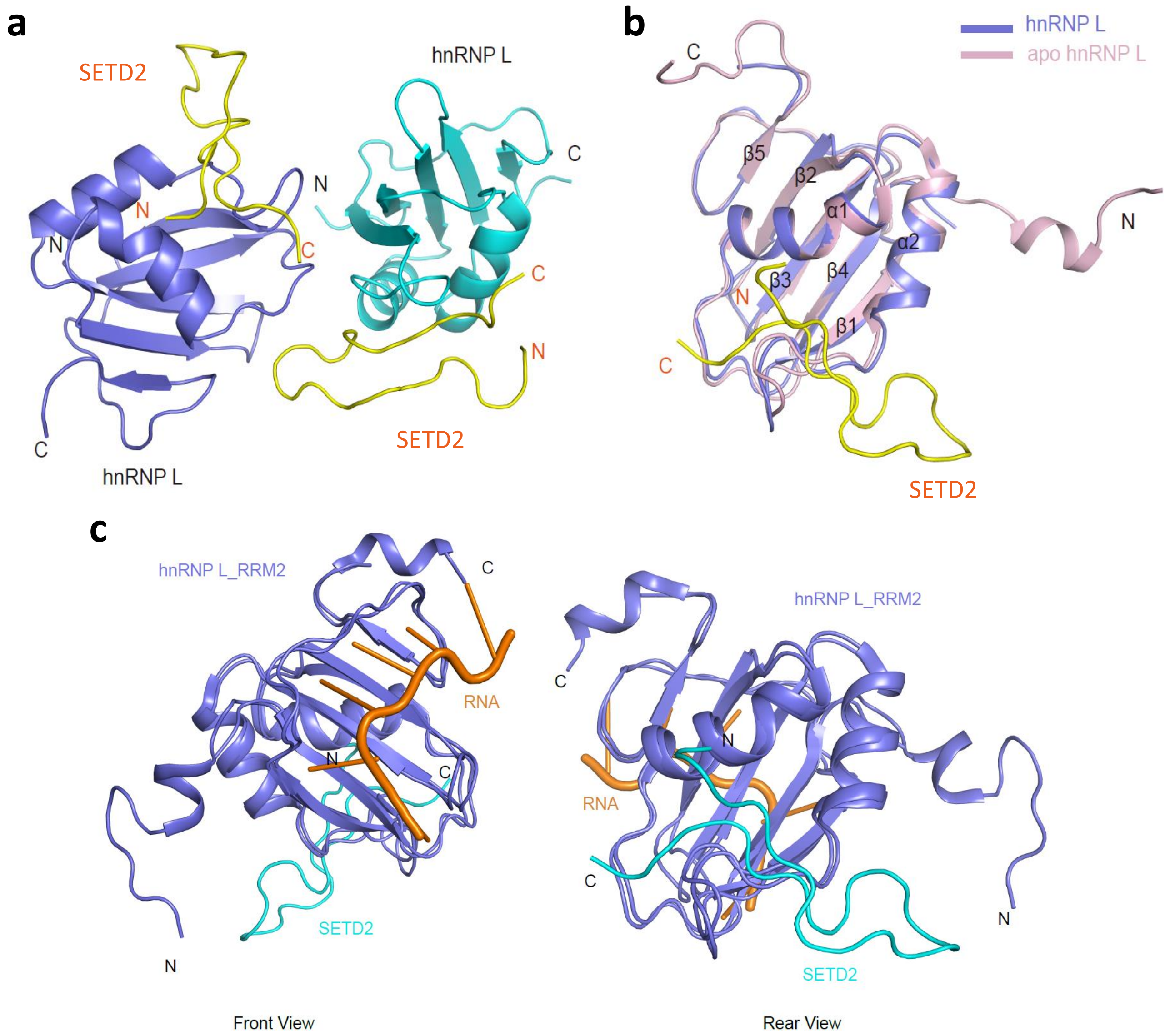

Supplementary Figure 1. **Structural studies reveal the mode of SETD2-hnRNP L interaction.** (a) Two highly similar hnRNP L RRM2 chains A (purple) and C (Cyan) in complex with SETD2$^{2167\text{-}2192}$ chains B and D (yellow), respectively. (b) Alignment of hnRNP L to apo hnRNP L reveals no significant structural changes upon SETD2$^{2167\text{-}2192}$ binding. (c) A superimposition of hnRNP L RRM1 in complex with RNA and RRM2 in complex with SETD2$^{2167\text{-}2192}$. The RRM-RNA model is the structure of hnRNP L RRM1 in complex with a 5'-CACAC-3' RNA sequence (PDB ID: 2MQO). Most of the RRMs bind to RNA in the same beta-sheet face but not dorsal helix face. The superimposition of the two structure clearly show that the peptide binding and RNA binding interfaces are separated.

a

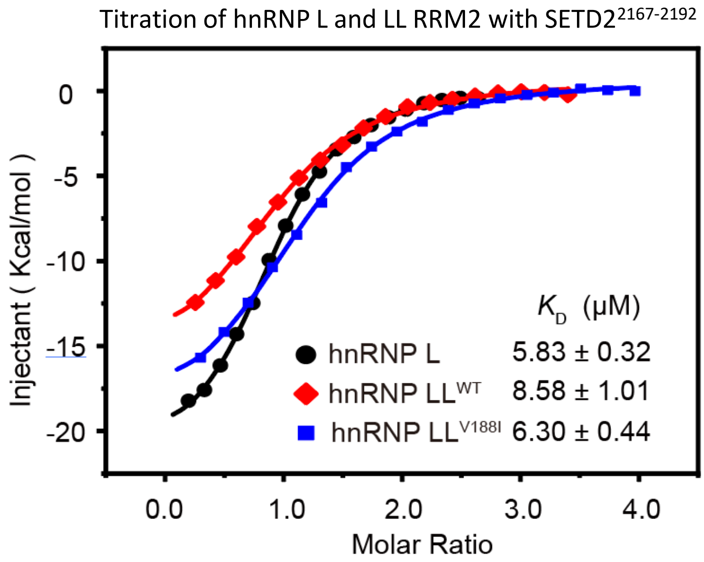

b

```
RRM4    --------------------RIQHPSNVLHFFN-APLEVTEENFFEICDELGVKRPSSVK    39
RRM2    ---------------------RSVNSVLLFTILNPIYSITTDVLYTICNPCGPVQR--IV    37
RRM1    -------------------------SPVVHIRGLIDGVVEADLV-EALQEFGPISY--VV    32
RRM3    PSRYGPQYGHPPPPPPPPEYGPHADSPVLMVYGLDQSKMNCDRVFNVFCLYGNVEK--VK    58
                                 * ::         :  : .       *      :

RRM4    VFSGKSERSSSGLLEWESKSDALETLGFLNHYQMKNPNGPYPYTLKLCF-----------    88
RRM2    IFRKN---GVQAMVEFDSVQSAQRAKASLNGA------DIYSGCCTLKIE---YAKPTRL    85
RRM1    VMPKK----RQALVEFEDVLGACNAVNYAADN-------------QIYIA----------    65
RRM3    FMKSK---PGAAMVEMADGYAVDRAITHLNNN------FMFGQKLNVCVSKQPAIMPGQS    109
        .:  :      .::*  .   . .:                     : .

RRM4    -------------------    88
RRM2    NVFK-NDQDTWDYTNP---    100
RRM1    -----GHPAFVNYSTSQKI    79
RRM3    YGLEDGSCSYKDFSES---    125
```

Supplementary Figure 2. **Residues in RRMs govern interaction with SETD2.** (a) ITC fitting curves of hnRNP L^{WT} (black), hnRNP LL^{WT} (red), hnRNP LL^{V188I} (blue), titrated with $SETD2^{2167\text{-}2192}$ peptide. (b) Sequence alignment of the RRMs of hnRNP L. Residues contributing to hydrophobic interactions and hydrogen bonding with SETD2 are highlighted in red and green, respectively.

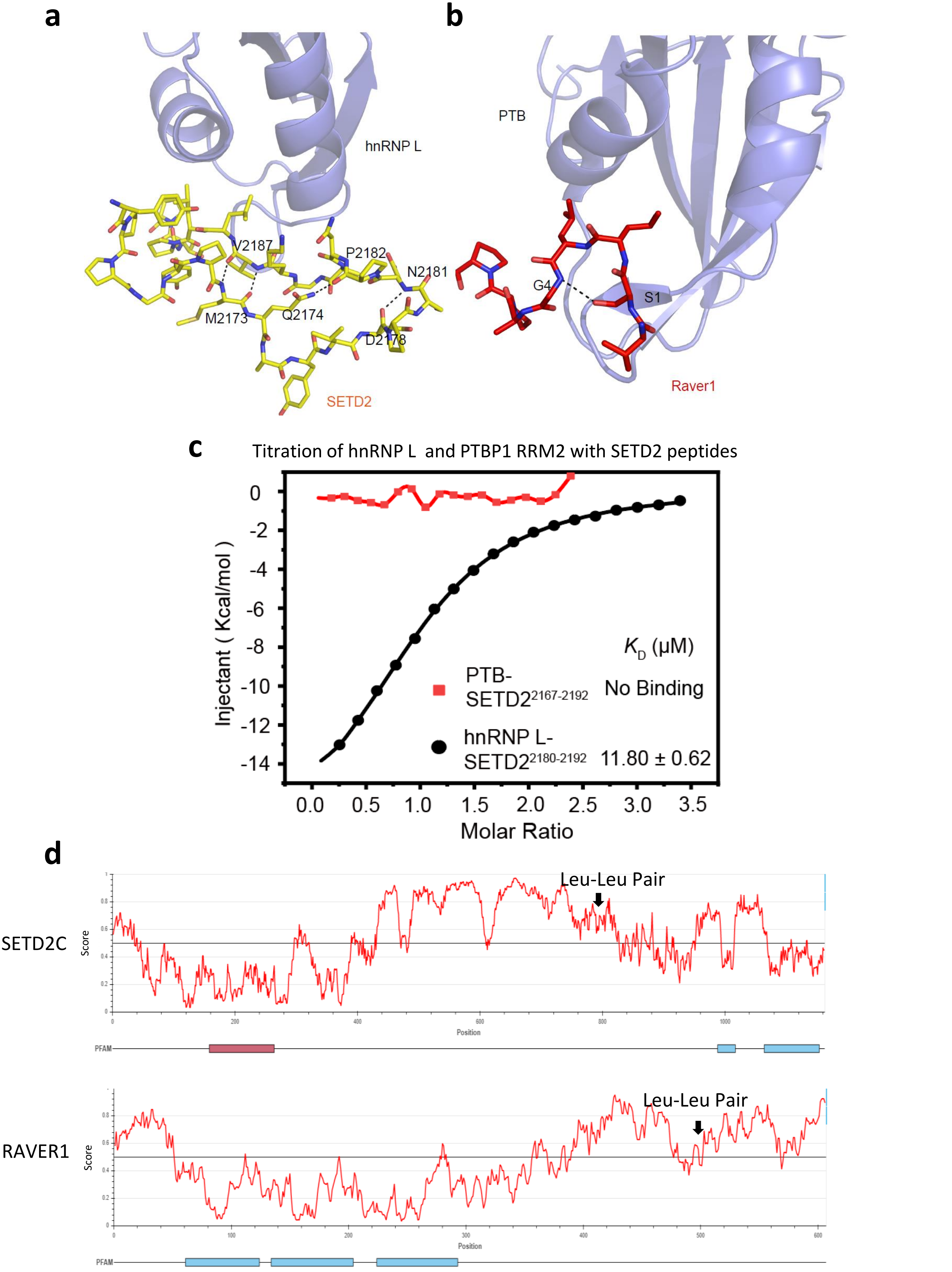

Supplementary Figure 3. **Raver1-PTBP1 and SETD2-hnRNP L interactions share similarities.** (a, b) Intramolecular interaction comparison of hnRNP L-SETD2$^{2167\text{-}2192}$ and PTB RRM2-PRI3. Hydrogen bonds are shown as black dashes. (c) ITC fitting curves of PTB RRM2 with SETD2$^{2167\text{-}2192}$ (red),hnRNP L with SETD2$^{2180\text{-}2192}$ (black). (d) IUPRED2 analysis showing disordered regions of SETD2C (1404-2564) and RAVER1.

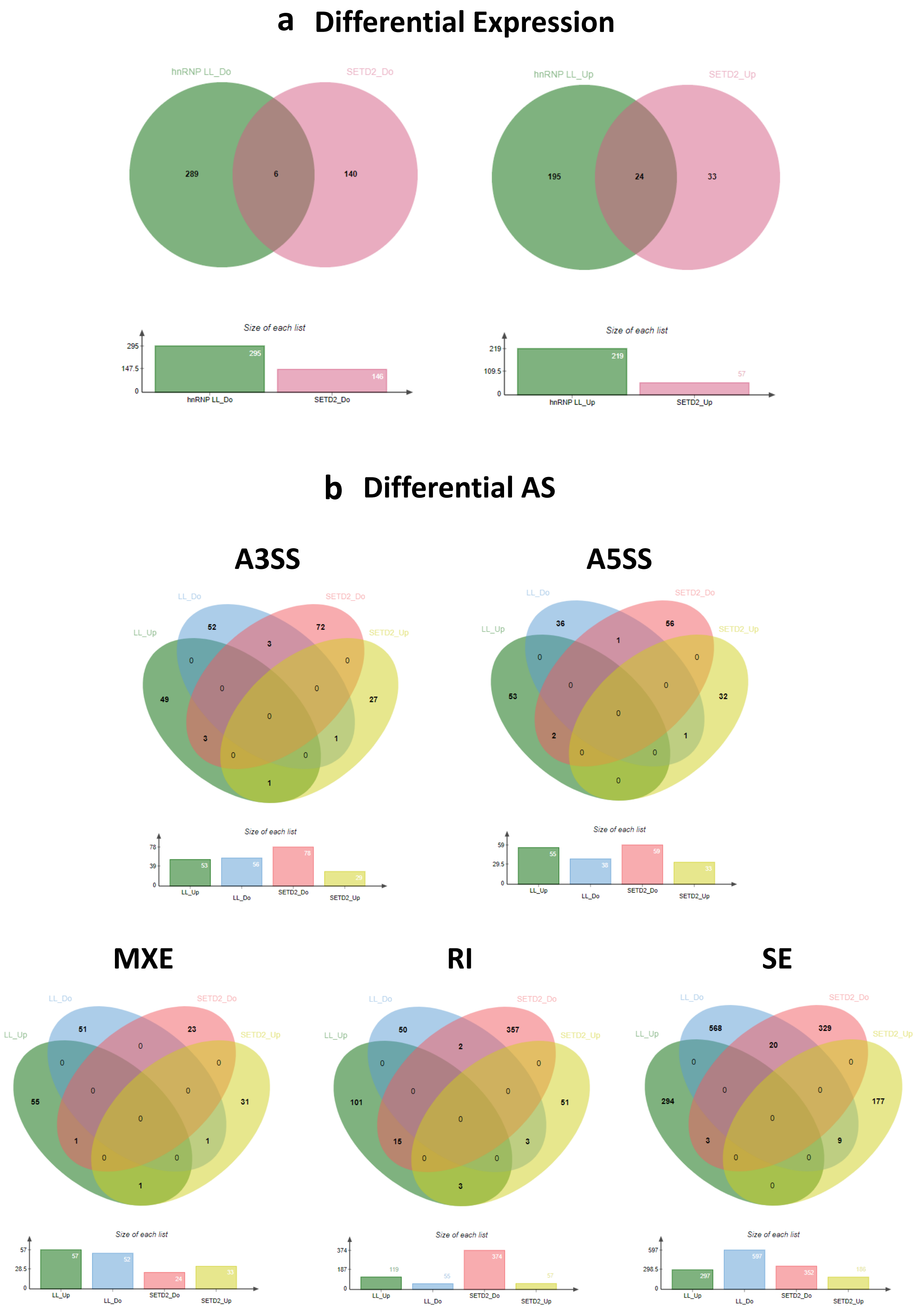

Supplementary Figure 4. **Overlap of transcriptome changes upon *setd2* and *hnrnpl* depletion is small in 293T cells.**(a) Venn diagram showing the overlap between differential gene expression and (b) AS events upon SETD2 and hnRNP LL depletion as compared to scramble siRNA treated cells. The overlap between different type of AS events are shown. A3SS-Alternate 3’ Splice Site, A5SS-Alternate 5’ Splice Site, MXE-Mutually Exclusive Exons, RI-Retained Intron, SE-Skipped Exon.

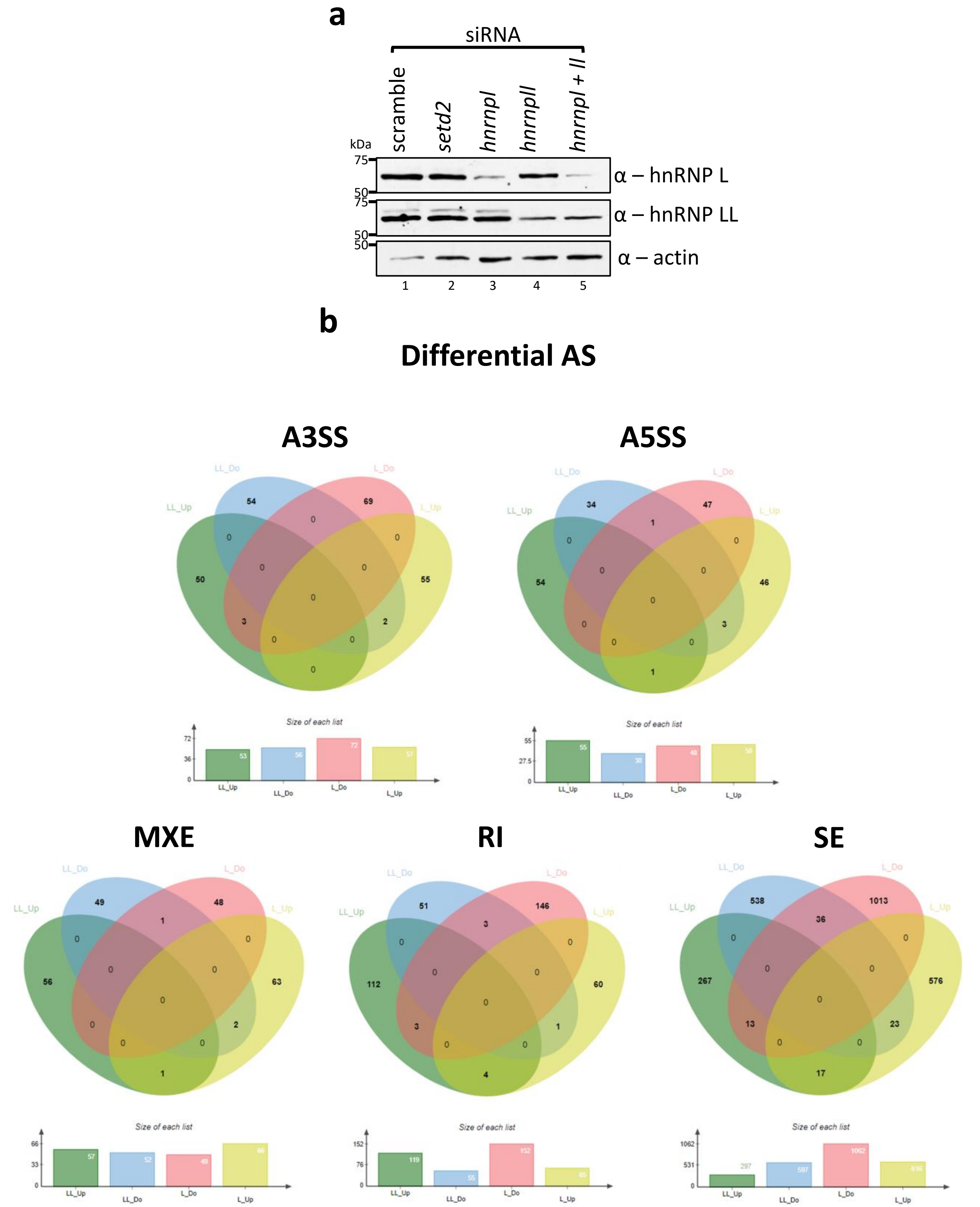

Supplementary Figure 5. **Depletion of *hnrnpl* and *hnrnpll* results in non-overlapping AS events.** (a) Western blotting of whole cell extracts probed with the depicted antibodies. The experiment was repeated 3 times all yielding similar results. Source data are provided as a Source Data File. (b) Venn diagram showing the overlap between differential AS events upon hnRNP L and hnRNP LL depletion as compared to scramble siRNA treated cells. The overlap between different type of AS events are shown. A3SS-Alternate 3' Splice Site, A5SS-Alternate 5' Splice Site, MXE-Mutually Exclusive Exons, RI-Retained Intron, SE-Skipped Exon.

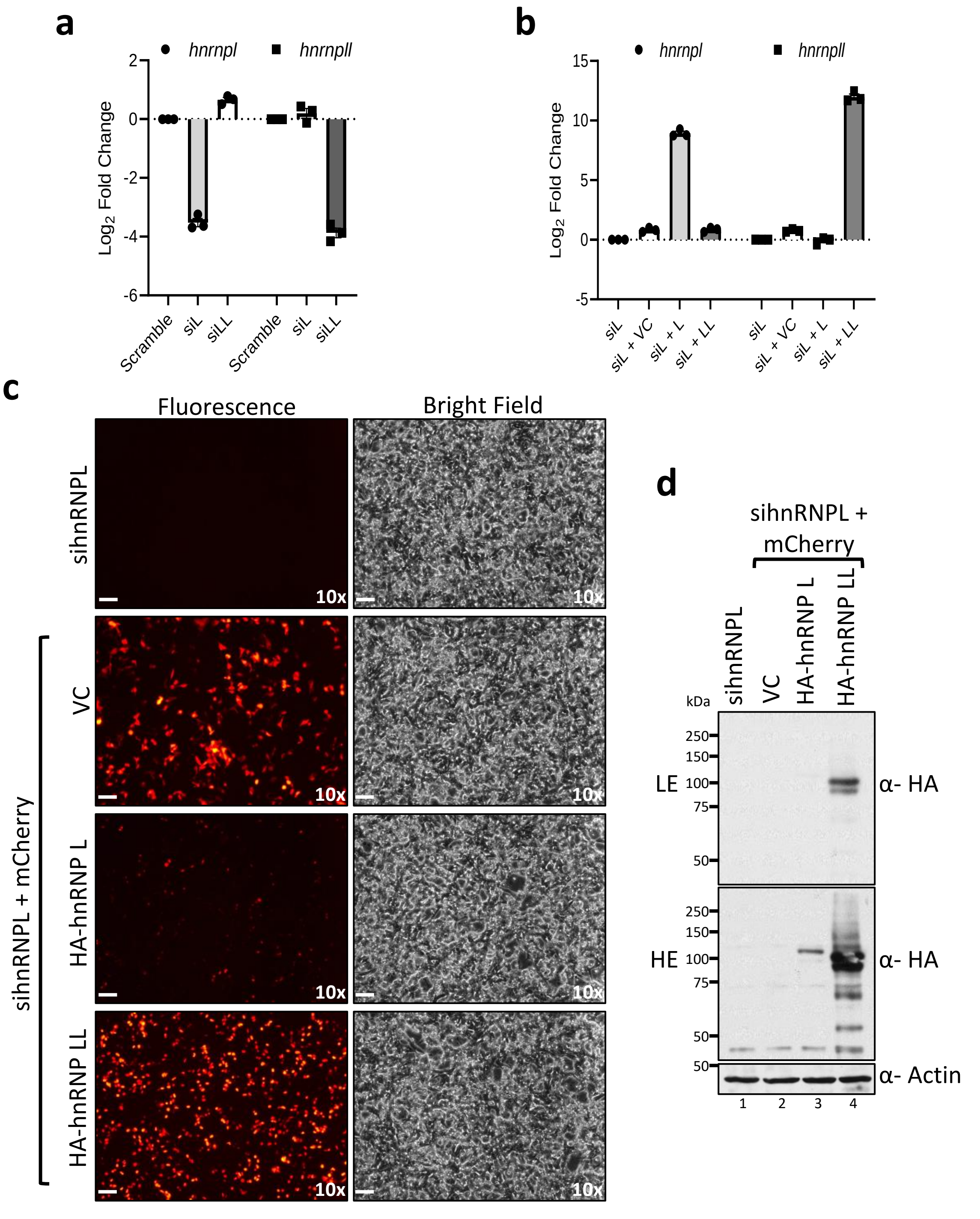

Supplementary Figure 6. **qPCR, microscopy and wester blotting demonstrates expected changes in the levels of *hnrnp*l and *hnrnpll*.** (a) RNA was isolated from 293T cells 72 hours post-transfection with scramble siRNA, or siRNA against *hnrnpl* or *hnrnpll*. Also, (b) RNA was isolated from sihnRNPL expressing 293T cells rescued with vector control (VC), hnRNP L or hnRNP LL constructs. For each sample n = 3 independent biological samples were examined in the same sequencing run. Data are presented as mean values with Standard Error of Mean. Unpaired t test (two-tailed) was performed. p-value <0.05 was considered significant. p-values are depicted on the top of the respective graphs. RNA with RIN number >8 was used. 18S rRNA was used for normalization. Specificity of the target was confirmed by a single sharp melt curve and resolving products on agarose gel. RNA was isolated using Qiagen Kit and subjected to DNaseI treatment for 30 mins at 37° C. (c) Microscopy images and (d) western blot showing the expression of mCherry-HA-hnRNP L/LL in sihnRNPL expressing cells. The lower expression of ectopically expressed hnRNP L as compared to LL is because the exogenous hnRNP L is not resistant to sihnRNPL. The experiment was repeated twice, both yielding similar results. Source data are provided as a Source Data File. The scale bar is 1 mm. LE-Lower Exposure, HE- Higher Exposure.

Protein	Cancer Type	Protein Change	Number of Samples
SETD2	Uterine Endometrioid Carcinoma	P2192H	1555
hnRNP L	Oligoastrocytoma	Y257C	27
hnRNP L	Head and Neck Squamous Cell Carcinoma	I214V	150

Supplementary Figure 7. **Residues important for SETD2-hnRNP L interaction are mutated in cancer.** Mutations in residues of SETD2 and hnRNP L found in cancer as per cBioPortal for Cancer Genomics (https://www.cbioportal.org/) that are important for SETD2-hnRNP L interaction.

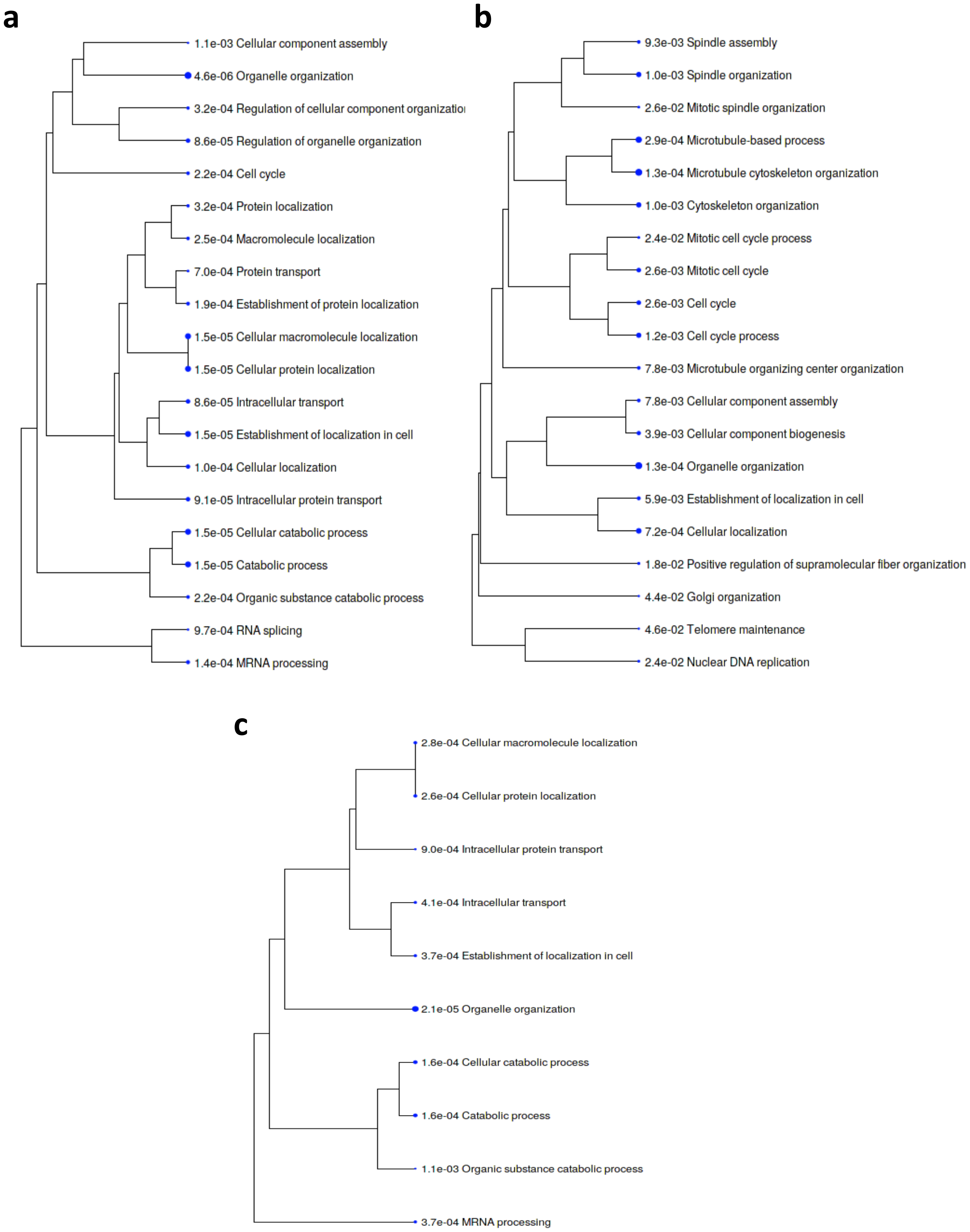

Supplementary Figure 8. **Different pathways are altered upon hnRNP L and LL depletion.** GO-term analysis using ShinyGo of differential AS events (a) upon hnRNP L depletion, (b) upon hnRNP LL depletion, and (c) unique to hnRNP L depletion as compared to hnRNP LL depletion.

Supplementary Table 1. ITC results

Protein	Peptide	ΔH	-TΔS	ΔG	N[a]	K_D[b] [μM]	K_D[c] (Mean) [μM]	Krel[d]
		[kcal/mol]						
hnRNP L	SETD2[2167-2192]	-20.1 ± 0.5	13.0	-7.02	0.93	5.83 ± 0.54	5.86 ± 0.05	1.0
		-22.4 ± 0.4	15.4	-7.02	0.92	5.83 ± 0.32		
		-20.9 ± 0.5	13.9	-7.02	0.93	5.91 ± 0.53		
	SETD2[2180-2192]	-19.1 ± 1.4	12.5	-6.63	1.01	11.40 ± 1.39	11.57 ± 0.21	2.0
		-19.2 ± 0.6	12.5	-6.61	1.00	11.80 ± 0.62		
		-19.1 ± 0.8	12.5	-6.63	0.96	11.50 ± 0.77		
hnRNP LL	SETD2[2167-2192]	-17.7 ± 0.8	10.9	-6.30	0.95	8.58 ± 1.01	8.27 ± 0.28	1.4
		-17.2 ± 0.8	10.4	-6.31	1.04	8.04 ± 0.98		
		-17.3 ± 0.3	10.5	-6.30	1.04	8.19 ± 1.10		
	SETD2[2180-2192]	-15.2 ± 1.0	8.9	-6.30	0.82	20.20 ± 1.96	20.05 ± 0.22	3.4
		-14.6 ± 1.5	8.3	-6.31	0.83	19.80 ± 2.98		
		-14.6 ± 2.4	8.3	-6.30	0.89	20.10 ± 5.10		
hnRNP L[V210A]	SETD2[2167-2192]	-13.5 ± 0.4	6.7	-6.76	0.93	9.10 ± 0.76	9.11 ± 0.22	1.6
		-13.1 ± 0.8	6.3	-6.75	0.78	8.90 ± 1.39		
		-12.0 ± 0.4	5.3	-6.78	1.13	9.33 ± 0.89		
hnRNP L[I214A]	SETD2[2167-2192]	-13.5 ± 0.7	7.1	-6.45	1.01	15.60 ± 1.95	16.03 ± 0.38	2.7
		-13.6 ± 1.2	7.2	-6.42	1.05	16.30 ± 3.42		
		-13.9 ± 0.5	7.4	-6.43	1.18	16.20 ± 1.41		
hnRNP L[I256A]	SETD2[2167-2192]	-10.8 ± 1.9	4.8	-5.98	1.30	34.80 ± 12.0	35.20 ± 0.53	6.0
		-11.7 ± 1.0	5.8	-5.96	1.31	35.80 ± 5.71		
		-11.8 ± 1.0	5.8	-5.98	1.18	35.00 ± 5.34		
hnRNP L[L263A]	SETD2[2167-2192]	-15.8 ± 1.1	9.3	-6.50	0.85	14.40 ± 2.29	13.73 ± 0.61	2.3
		-14.7 ± 1.2	8.2	-6.55	0.98	13.60 ± 2.74		
		-16.4 ± 1.1	9.9	-6.63	0.93	13.20 ± 2.21		
hnRNP LL[V188I]	SETD2[2167-2192]	-20.2 ± 0.7	13.2	-6.99	1.10	6.16 ± 0.60	6.30 ± 0.14	1.1
		-22.0 ± 0.7	15.0	-6.96	0.96	6.44 ± 0.56		
		-20.6 ± 0.5	13.6	-6.98	1.12	6.30 ± 0.44		
hnRNP L[Y257A]	SETD2[2167-2192]				N.D.[e]			
hnRNP L	SETD2[2113-2140]				N.D.			
hnRNP L	SETD2[L2188A]				N.D.			
hnRNP L	SETD2[L2189A]				N.D.			
PTB1	SETD2[2167-2192]				N.D.			

[a]N refers to the stoichiometric ratio of SETD2 peptides to hnRNP L.

[b]The fitting binding disassociation parameter of each independent titration.

[c]Reported error is the standard deviation of three independent experiments.

[d]Krel refers to the K_D relative to the K_D of SETD2[2167-2192] binding with hnRNP L.

[e]N.D., No detectable interaction under the experiment conditions.

Supplementary Table 2. ITC curves

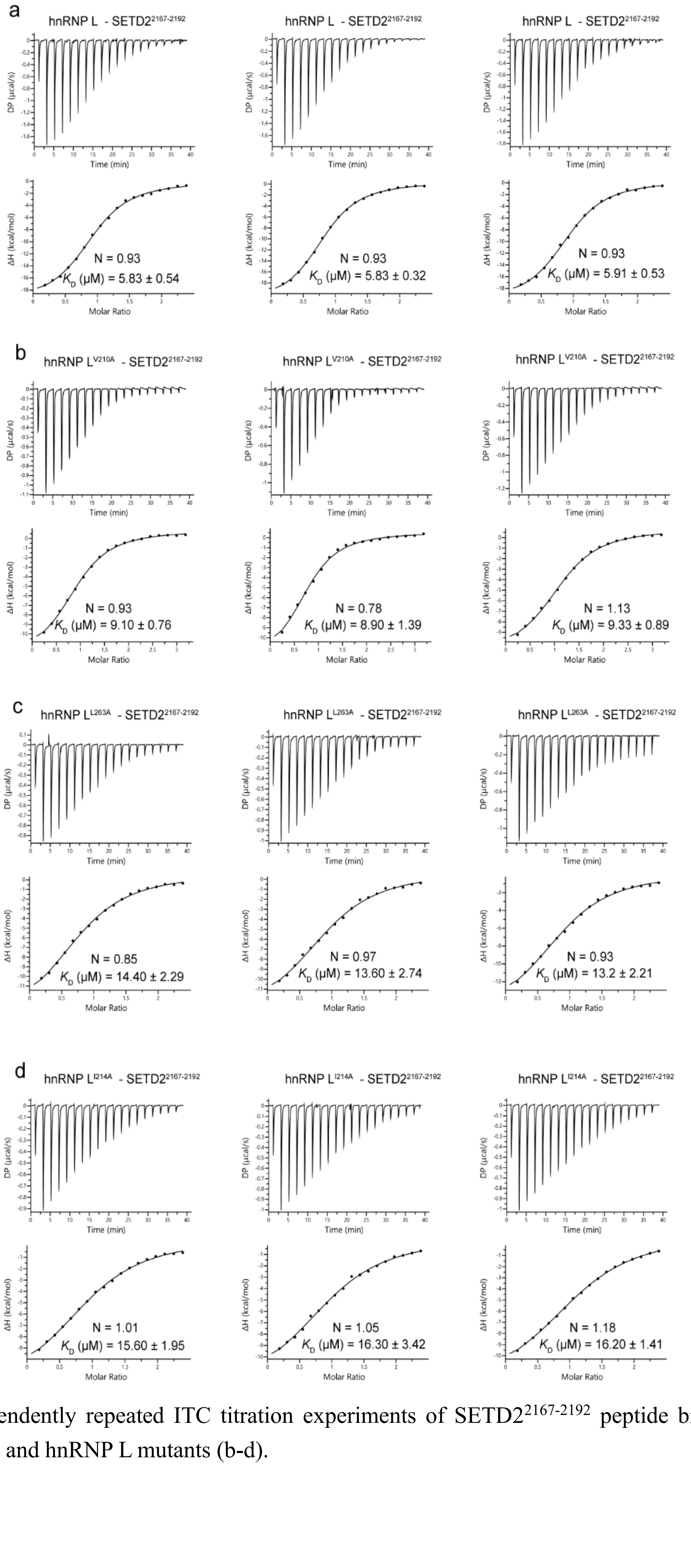

Three independently repeated ITC titration experiments of SETD2$^{2167\text{-}2192}$ peptide binding with hnRNP L (a) and hnRNP L mutants (b-d).

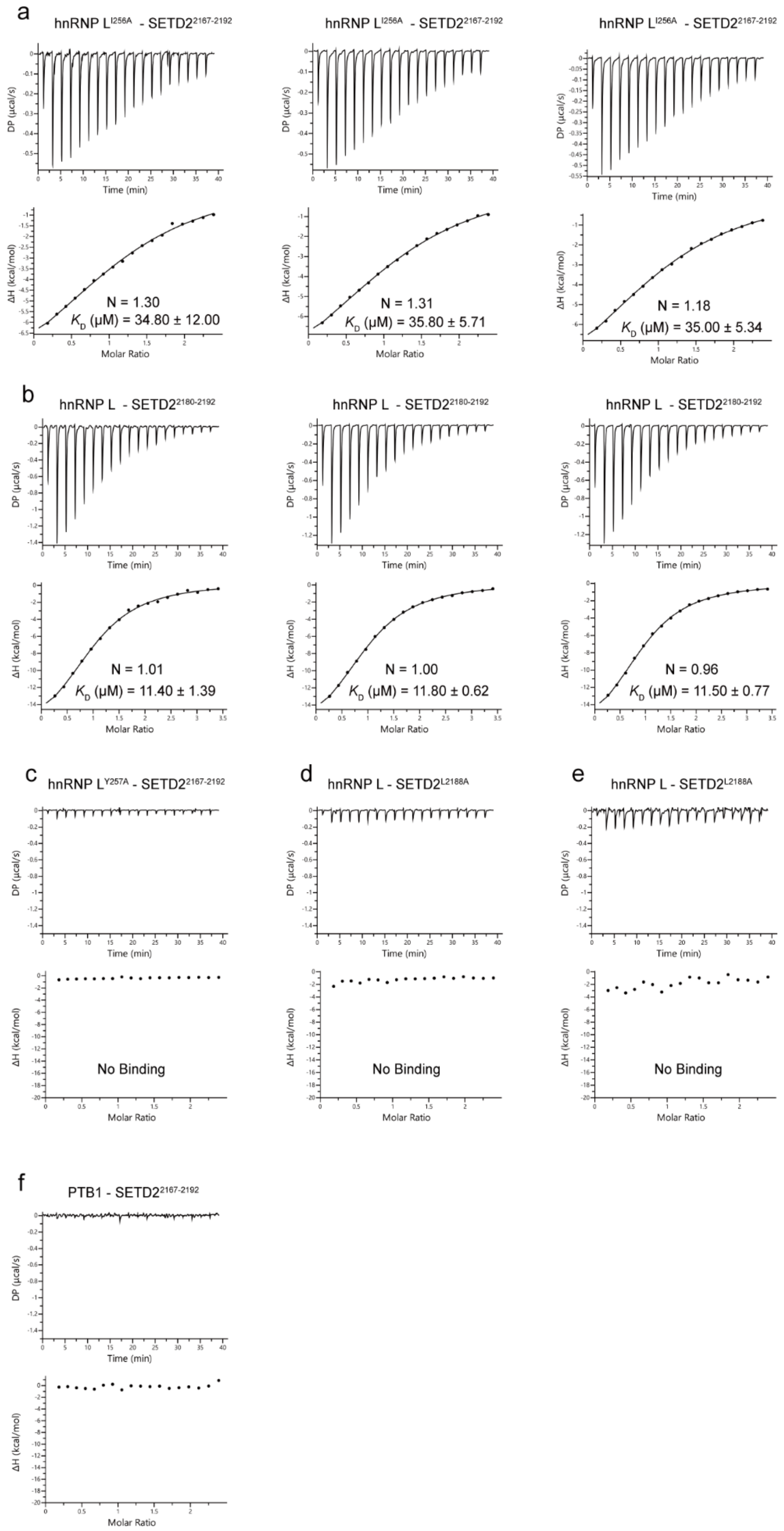

(a) ITC results of hnRNP L^{I256A} with $SETD2^{2167\text{-}2192}$ peptide. (b) ITC results of hnRNP L with $SETD2^{2180\text{-}2192}$ peptide. (c) ITC results of hnRNP L^{Y257A} with $SETD2^{2167\text{-}2192}$ peptide. (d, e) ITC results of hnRNP L with SETD2 mutants. (f) ITC results of PTB1 with $SETD2^{2167\text{-}2192}$ peptide.

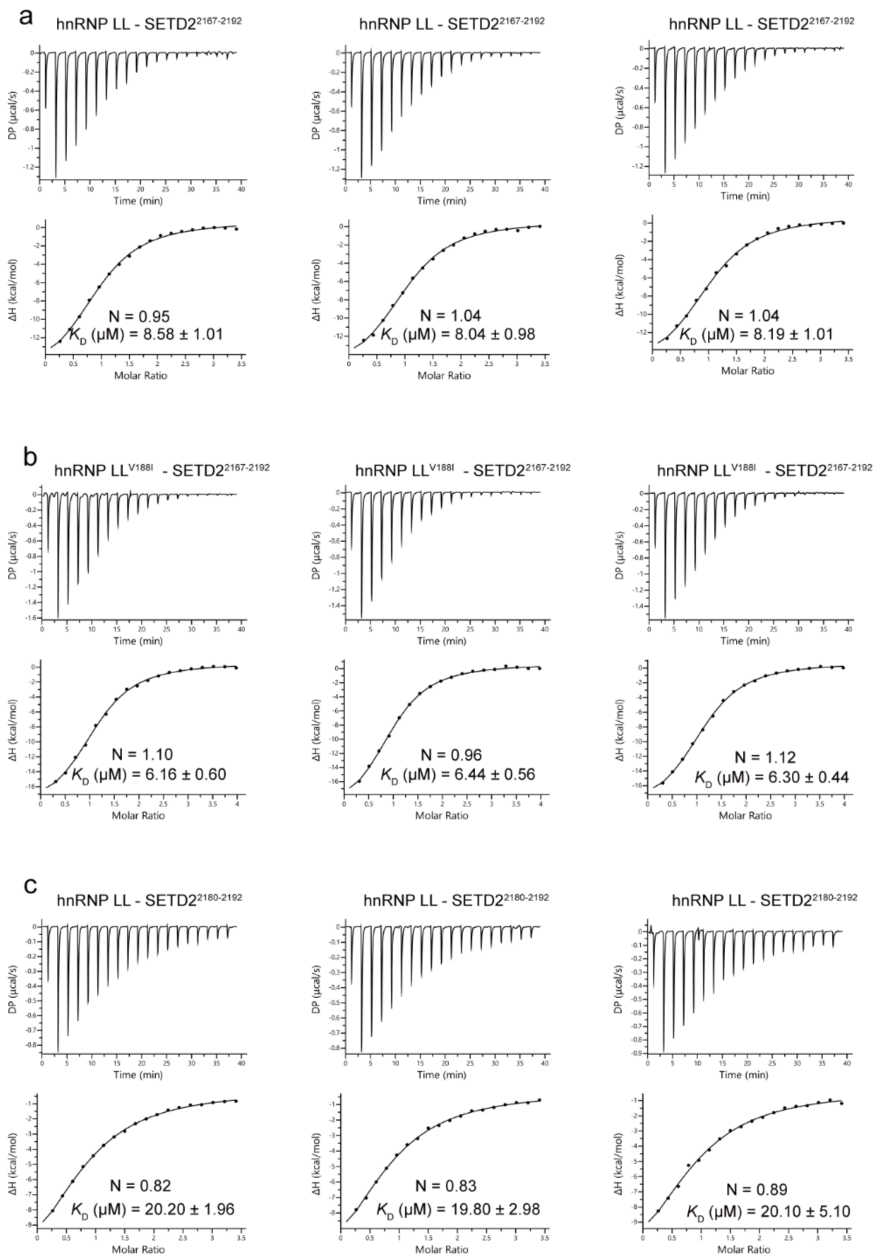

Three independently repeated ITC titration experiments of SETD2$^{2167\text{-}2192}$ peptide binding with hnRNP LL(a) and hnRNP LL^{V188I}(b) and experiments of SETD2$^{2180\text{-}2192}$ peptide binding with hnRNP LL(c).

Supplementary Table 3. Data collection, and refinement statistics of crystal data

	hnRNP L-SETD2$^{2167\text{-}2192}$	hnRNP LL-SETD2$^{2180\text{-}2192}$
Wavelength(Å)	0.979	0.979
Space group	$P2_1$	$P1$
Cell parameters		
a, b, c (Å)	56.78, 38.79, 68.98	41.68, 41.69, 42.64
α, β, γ (°)	90, 100.31, 90	85.02, 89.57, 71.12
Resolution(Å)	40.00-1.80(1.83-1.80)[a]	40.00-1.60(1.63-1.60)[a]
R_{merge} (%)	12.7(46.6)	6.3(31.4)
$CC_{1/2}$(%)	98.7(91.5)	99.1(90.8)
$I/\sigma I$	14.2(2.5)	16.8(3.5)
Completeness (%)	99.7(97.8)	96.6(94.3)
Average redundancy	5.2(4.9)	3.4(3.3)
**Refinement**		
No. reflections (overall)	27681	34230
No. reflections (test set)	1373	1732
R_{work}/R_{free}(%)	15.82/19.79	17.75/20.35
Number of atoms		
Protein	1553	1561
Ligand	394	177
H_2O	332	352
B factors (Å^2)		
Protein	17.89	16.25
Ligand	17.25	14.09
H_2O	30.49	28.10
r.m.s. deviations		
Bond lengths (Å)	0.006	0.006
Bond angles (°)	0.788	0.925
Ramachandran plot % residues[b]		
Favored	97.53	98.17
Allowed	2.47	1.83
Outliers	0	0

[a] Values in parentheses are for highest-resolution shell.

[b] Ramachandran plot analysis of model using RAMPAGE supported in Phenix suite.

Oligo	Sequence (5’-3’)
hnRNPL_F	TTCTGCTTATATGGCAATGTGG
hnRNPL_R	GACTGACCAGGCATGATGG
GAPDH _F	TTCGACAGTCAGCCGCATCTTCTT
GAPDH _R	CAGGCGCCCAATACGACCAAATC
hnRNPLL_F	AAAAGGATCACTCGGCCAGG
hnRNPLL_R	TTGTCTCTGGCGACCCTTTC
TJP1_e21_F	TGAAGGTATCAGCGGAGGGA
TJP1_e21_R	TCACGCAGTTACGAGCAAGT
TJP1_e20_F	GAGCTGCCTCAGTACTTGGT
TJP1_e20_R	TCCAGTCCCTTACCTTTCGC
TJP1_e19-21_F	AGACTCCCCTGGATTTAAGC
TJP1_e19-21_R	TCCGTATAGCTTGAGGACTCG
BPTF_e18_F	GCCGACAGTGATTGCAACTT
BPTF_e18_R	AACTGAGCCACTTATGGGGG
BPTF_e18a_F	AGGCAATCCAGTCACTATGGC
BPTF_e18a_R	CCTTGTGTTGCTGGCACATTT
BPTF_e19_R	TGAATTGCTTGTGGTTCCTCC

Supplementary Table 4. Sequence of oligos used to perform RT-PCR.
