# Supplementary material for: Structural basis of the interaction between SETD2 methyltransferase and hnRNP L paralogs for governing co-transcriptional splicing
Source: Nat Commun. 2021 Nov 8;12:6452. doi: 10.1038/s41467-021-26799-3 (PMC8575775; doi:10.1038/s41467-021-26799-3)
Supplement: Supplementary file 3 — Description of Additional Supplementary Files [file 41467_2021_26799_MOESM3_ESM.pdf]

Description of Additional Supplementary Files

File Name: Supplementary Data 1
Description: RNA-seq Differential Expression Analysis Report for hnRNP LL depleted cells vs WT

File Name: Supplementary Data 2
Description: SE changes in hnRNP L depleted cells vs WT

File Name: Supplementary Data 3
Description: Proteins identified by using SETD2C and its mutants as bait
