# Supplementary material for: Structural basis of the interaction between SETD2 methyltransferase and hnRNP L paralogs for governing co-transcriptional splicing
Source: Nat Commun. 2021 Nov 8;12:6452. doi: 10.1038/s41467-021-26799-3 (PMC8575775; doi:10.1038/s41467-021-26799-3)
Supplement: Supplementary file 5 — Reporting Summary [file 41467_2021_26799_MOESM5_ESM.pdf]

nature research

Corresponding author(s): Jerry Workman

Last updated by author(s): October 14, 2021

Reporting Summary

Nature Research wishes to improve the reproducibility of the work that we publish. This form provides structure for consistency and transparency in reporting. For further information on Nature Research policies, see our Editorial Policies and the Editorial Policy Checklist.

Statistics

For all statistical analyses, confirm that the following items are present in the figure legend, table legend, main text, or Methods section.

n/a	Confirmed	
☐	☒	The exact sample size (*n*) for each experimental group/condition, given as a discrete number and unit of measurement
☐	☒	A statement on whether measurements were taken from distinct samples or whether the same sample was measured repeatedly
☐	☒	The statistical test(s) used AND whether they are one- or two-sided<br>*Only common tests should be described solely by name; describe more complex techniques in the Methods section.*
☒	☐	A description of all covariates tested
☒	☐	A description of any assumptions or corrections, such as tests of normality and adjustment for multiple comparisons
☐	☒	A full description of the statistical parameters including central tendency (e.g. means) or other basic estimates (e.g. regression coefficient) AND variation (e.g. standard deviation) or associated estimates of uncertainty (e.g. confidence intervals)
☐	☒	For null hypothesis testing, the test statistic (e.g. F, t, r) with confidence intervals, effect sizes, degrees of freedom and P value noted<br>*Give P values as exact values whenever suitable.*
☒	☐	For Bayesian analysis, information on the choice of priors and Markov chain Monte Carlo settings
☒	☐	For hierarchical and complex designs, identification of the appropriate level for tests and full reporting of outcomes
☒	☐	Estimates of effect sizes (e.g. Cohen's *d*, Pearson's *r*), indicating how they were calculated

Our web collection on statistics for biologists contains articles on many of the points above.

Software and code

Policy information about availability of computer code

Data collection	Mass spectrometer scan functions and HPLC solvent gradients were controlled by the XCalibur 2.0.7 data system (Thermo Scientific). RNA-Seq samples (paired end reads 75 bp) were run on the Illumina NextSeq 500, using NextSeq Control Software 2.2.0.4, with NextSeq RTA 2.4.11. X-ray diffraction data sets of the crystals were collected at beamline 19U1 at the Shanghai Synchrotron Radiation Facility (SSRF) with a diffraction wavelength of 0.979 Å.
Data analysis	Mass spectrometry RAW files were extracted into .ms2 file format using RawDistiller v. 1.0. MS/MS spectra were first searched using ProLuCID. DTASelect v.1.9 44 was used to select and sort peptide/spectrum matches (PSMs) passing the following criteria set: PSMs were only retained if they had a DeltCn of at least 0.08; minimum XCorr values of 2.1 for singly-, 2.7 for doubly-, and 3.2 for triply-charged spectra; peptides had to be at least 7 amino acids long. Results from each sample were merged and compared using CONTRAST v.1.9. Combining all replicates, proteins had to be detected by at least 2 peptides and/or 2 spectral counts. Proteins that were subsets of others were removed using the parsimony option in DTASelect v.1.9 44 on the proteins detected after merging all runs. NSAF7 was used to create the final reports on all detected peptides and non-redundant proteins identified across the different runs. QPROT v. 1.2.2 was used to calculate a log fold change and Z-score for the samples compared to the mock control. proteins with log fold change >1 and Z-score > 2 were further analyzed in Ingenuity Pathway Analysis (IPA, Qiagen) to determine pathways enriched by the bait proteins.<br>The integrated heat data obtained by performing isothermal titration calorimetry were analyzed using a one-site binding model by MicroCal PEAQ 1.0.0.1259-ITC Analysis Software provided by the manufacturer.<br>The structure of hnRNP L/LL-SETD2 complex was determined by molecular replacement with the MOLREP 13.07.2020 program. The model was further built and refined using Coot 0.9.671 and Phenix.refine 1.19_409272-74, respectively. All the structures in the figures were generated using PyMOL 0.99rc6.<br>For nucleic acid sequencing data, Raw reads were demultiplexed into FASTQ format allowing up to one mismatch using Illumina bcl2fastq2 v2.18. RNA seq reads were aligned to the human genome (hg38 and Ensembl 96 gene models) using STAR (version STAR_2.7.3a). TPM expression values were generated using RSEM (version v1.3.0). edgeR (version 3.24.3 with R 3.5.2) was applied to perform differential expression analysis, using only protein-coding and lncRNA genes. To perform differential splicing analysis, we used rMATs (version 4.0.2) with

default parameters starting from FASTQ files.

For manuscripts utilizing custom algorithms or software that are central to the research but not yet described in published literature, software must be made available to editors and reviewers. We strongly encourage code deposition in a community repository (e.g. GitHub). See the Nature Research guidelines for submitting code & software for further information.

Data

Policy information about availability of data

All manuscripts must include a data availability statement. This statement should provide the following information, where applicable:

- Accession codes, unique identifiers, or web links for publicly available datasets
- A list of figures that have associated raw data
- A description of any restrictions on data availability

All relevant data are available. The RNA-data sets are available in the Gene Expression Omnibus (GEO) database under the accession number GSE174426 (https://www.ncbi.nlm.nih.gov/geo/query/acc.cgi?acc=GSE174426). The mass spectrometry proteomics data is available at the ProteomeXchange Consortium via the PRIDE partner repository with the dataset identifiers PXD019376 (http://proteomecentral.proteomexchange.org/cgi/GetDataset?ID=PXD019376), PXD022946 (http://proteomecentral.proteomexchange.org/cgi/GetDataset?ID=PXD022946), and PXD025942 (http://proteomecentral.proteomexchange.org/cgi/GetDataset?ID=PXD025942). The hnRNP L-SETD2 and the hnRNP LL-SETD2 complexes have been deposited to PDB with entry ID: 7EVR (https://www.rcsb.org/structure/7EVR) and 7EVS (https://www.rcsb.org/structure/7EVS), respectively. The structure of PTB1-PRI3 with PDB ID: 3ZZY(https://www.rcsb.org/structure/3ZZY) was used as the search model for determining hnRNP L-SETD2 complex.

Field-specific reporting

Please select the one below that is the best fit for your research. If you are not sure, read the appropriate sections before making your selection.

☒ Life sciences ☐ Behavioural & social sciences ☐ Ecological, evolutionary & environmental sciences

For a reference copy of the document with all sections, see nature.com/documents/nr-reporting-summary-flat.pdf

Life sciences study design

All studies must disclose on these points even when the disclosure is negative.

Sample size	Our study did not involve any organisms or human samples or clinical research. Hence, no sample size calculation was performed. Two independent biological replicates were used in high throughput experiments. As the replicates were in very good agreement with one another, hence, the sample size was considered sufficient.
Data exclusions	No data was excluded.
Replication	Experiments were replicated by using biologically independent replicates at least twice and by utilizing more than one technique to confirm the findings. For instance, crucial mass spectrometry findings were also confirmed by western blotting of the purified complexes. Depletion of the target protein was confirmed by western blotting, RT-PCR before proceeding for high through-put sequencing. All attempts at replication were successful.
Randomization	The interpretation of the experiments performed by us required comparison of data from experimental sets versus the control set. For instance, for mass spectrometry experiments, data from known samples of interest were compared against mock samples. For RNA-Seq experiments, known samples of interest were first compared against known scramble siRNA treated cells. Hence, randomization was not possible.
Blinding	The interpretation of the experiments performed by us required comparison of data from experimental sets versus the control set. For instance, for mass spectrometry experiments, data from known samples of interest were compared against mock samples. For RNA-Seq experiments, known samples of interest were first compared against known scramble siRNA treated cells. Hence, the identity of the control and the experimental sets were required to be known.

Reporting for specific materials, systems and methods

We require information from authors about some types of materials, experimental systems and methods used in many studies. Here, indicate whether each material, system or method listed is relevant to your study. If you are not sure if a list item applies to your research, read the appropriate section before selecting a response.

Materials & experimental systems

n/a	Involved in the study
☐	☒ Antibodies
☐	☒ Eukaryotic cell lines
☒	☐ Palaeontology and archaeology
☒	☐ Animals and other organisms
☒	☐ Human research participants
☒	☐ Clinical data
☒	☐ Dual use research of concern

Methods

n/a	Involved in the study
☒	☐ ChIP-seq
☒	☐ Flow cytometry
☒	☐ MRI-based neuroimaging

Antibodies

Antibodies used

hnRNP L (CST 37562, dilution 1:3000), hnRNP LL (CST 4783S, dilution 1:3000), FLAG (Sigma-Aldrich A8592, dilution 1:10000), Pol II Ser2P (Abcam ab5095, dilution 1:5000), Halo (Promega G9211, dilution 1:10000), SETD2 (Aviva OAEB00589, dilution 1:3000), HA (Sigma 04-902, dilution 1:10000), β-actin (Abcam ab8224, dilution 1:2500).

Validation

Commercially available antibodies were used in the study that are validated as per published reports and the manufacturer. Further validation was performed by us as follows:
1) hnRNP L and LL: Depletion of hnRNP L and LL resulted in the decreased intensity of their respective bands [Supplementary Figure 5a]. Product website for hnRNP L (https://www.cellsignal.com/products/primary-antibodies/hnrnp-l-antibody/37562) states "Specificity / Sensitivity: hnRNP L Antibody recognizes endogenous levels of total hnRNP L protein. Also product website for hnRNP LL (https://www.cellsignal.com/products/primary-antibodies/hnrnp-ll-antibody/4783) shows western blot analysis of extracts from Hep G2 and HeLa cells using hnRNP LL Antibody and states "Sensitivity" as Endogenous.
Species Reactivity: Human, Mouse, Rat, Monkey."
2) FLAG, HA: Probing lysates of untrasfected mammalian cells and untransformed bacterial cells do not show bands. The transfected/transformed cells show band of the expected size. FLAG: Product website (https://www.sigmaaldrich.com/catalog/product/sigma/a8592?lang=en&region=US) states that the Monoclonal ANTI-FLAG M2-Peroxidase is a mouse IgG antibody covalently conjugated to horseradish peroxidase (HRP). The antibody binds to FLAG fusion proteins and recognizes the FLAG epitope at N-terminal, Met-N-terminal, C-terminal, and internal FLAG peptides. HA: Product website (https://www.sigmaaldrich.com/catalog/product/mm/04902?lang=en&region=US) states that the antibody recognizes recombinant proteins containing the HA epitope tag.
4) Pol II Ser2P: This antibody has been successfully used by us to demonstrate that SETD2 lacking the SRI domain loses interaction with RNA Pol II. Mass spectrometry data of the purified complexes validate these findings (Figure 3). Product website (https://www.abcam.com/rna-polymerase-ii-ctd-repeat-ysptsps-phospho-s2-antibody-ab5095.html) states "Specificity: This antibody recognises the phosphorylated serine found in the amino acid 2 position of the C-terminal domain repeat YSPTSPS. Species reactivity: Mouse, Rat, Human, Saccharomyces cerevisiae."
5) Halo: The product page (https://www.promega.com/products/protein-detection/primary-and-secondary-antibodies/anti-halotag-monoclonal-antibody/?catNum=G9211) states that the antibody has little to no cross-reactivity with other non-HaloTag® proteins. In our experiments, the antibody always gives intense band at the expected molecular weight. This antibody has been previously used by us in the paper "The disordered regions of the methyltransferase SETD2 govern its function by regulating its proteolysis and phase separation. J. Biol. Chem. 297, 101075 (2021)."
6) SETD2: As per the product page (https://www.avivasysbio.com/goat-anti-hypb-setd2-internal-region-antibody-oaeb00589.html), the antibody was developed using the peptide C-ERDPDKQTQNKE as immunogen which is specific to SETD2. This antibody has been previously used by us in the paper "The methyltransferase SETD2 couples transcription and splicing by engaging mRNA processing factors through its SHI domain. Nat Commun. 12, 1443 (2021) 33664260". Species Reactivity: Human, Mouse, Dog, Pig.
7) Actin: The product page (https://www.abcam.com/beta-actin-antibody-mabcam-8224-loading-control-ab8224.html) states that the antibody recognises a single band at 42kD representing beta Actin. The immunogen used for this product shares 77% homology with Gamma actin/actin cytoplasmic. It also describes species reactivity as Mouse, Rat, Human, Xenopus laevis, Drosophila melanogaster, Schizosaccharomyces pombe.

Eukaryotic cell lines

Policy information about cell lines

Cell line source(s)	HEK293T from ATCC
Authentication	STR analysis
Mycoplasma contamination	All cell lines tested negative for mycoplasma contamination.
Commonly misidentified lines (See ICLAC register)	No such cell lines were used in the study.
