# Supplementary material for: Structural basis of the interaction between SETD2 methyltransferase and hnRNP L paralogs for governing co-transcriptional splicing
Source: Nat Commun. 2021 Nov 8;12:6452. doi: 10.1038/s41467-021-26799-3 (PMC8575775; doi:10.1038/s41467-021-26799-3)

Figure 1

Figure 1e

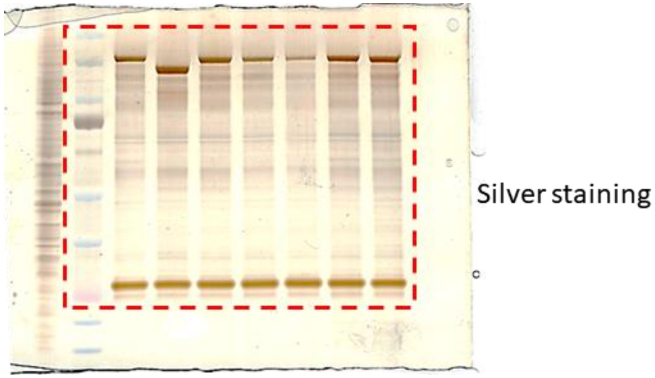

Figure 1f

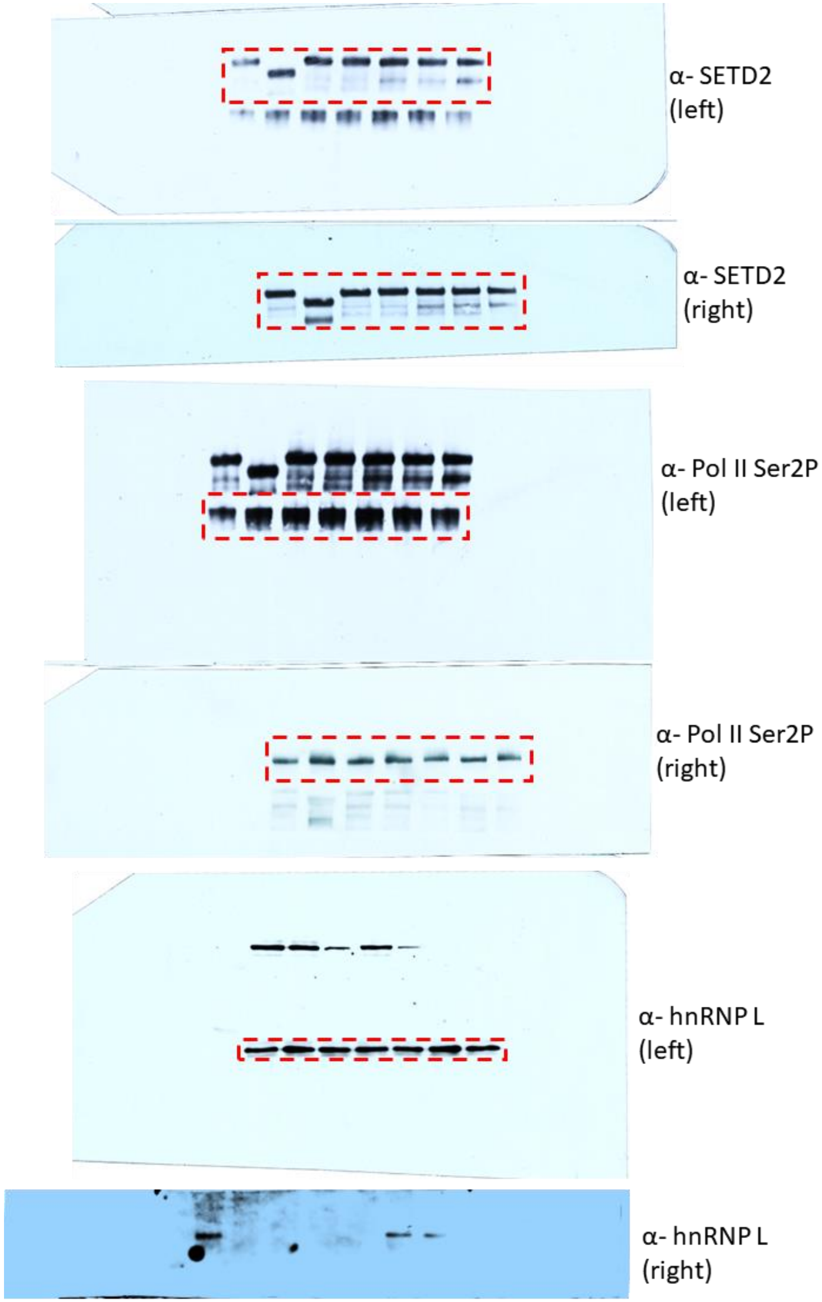

Figure 3

Figure 3e

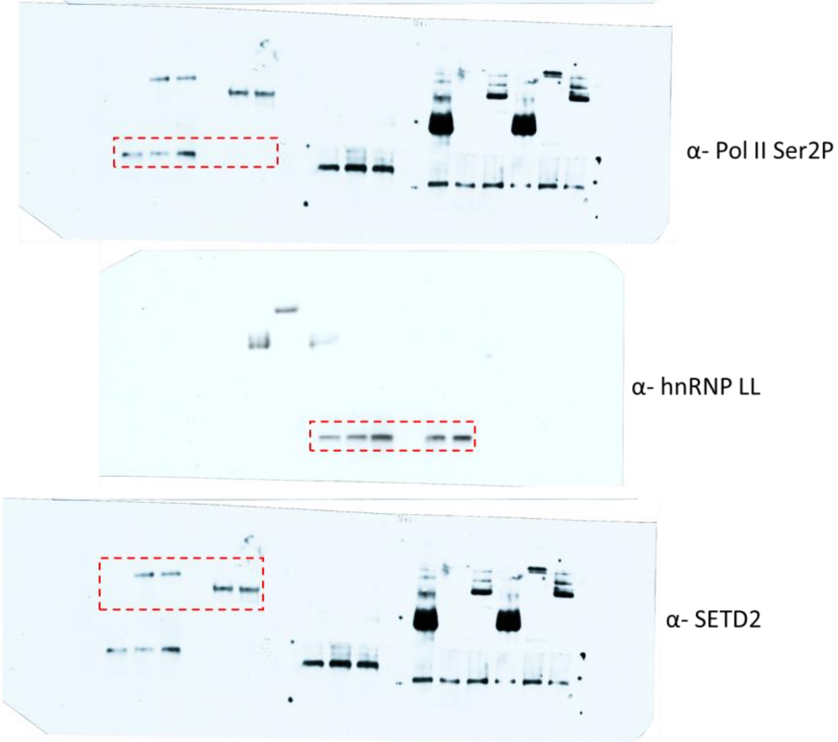

Figure 3f

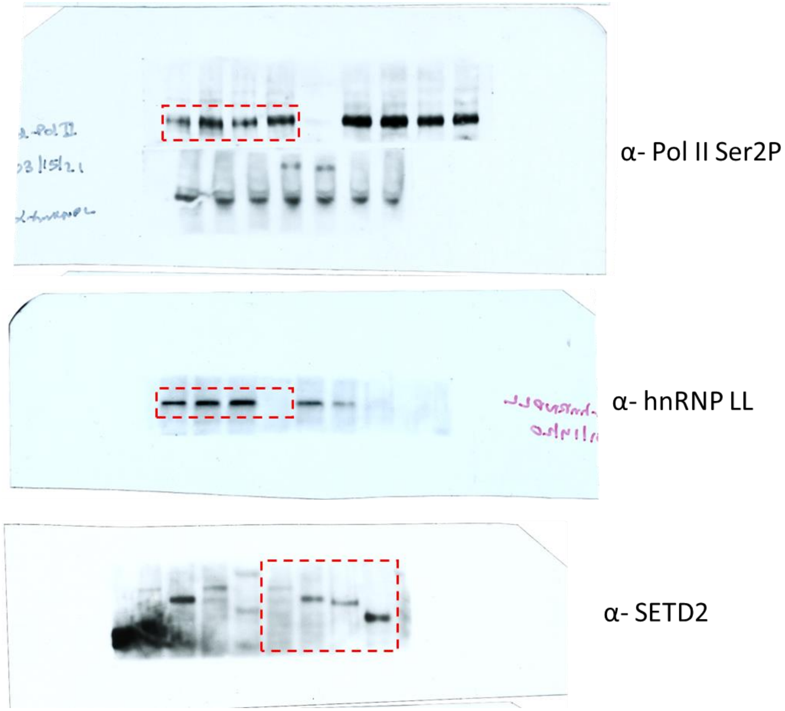

Figure 3

Figure 3j

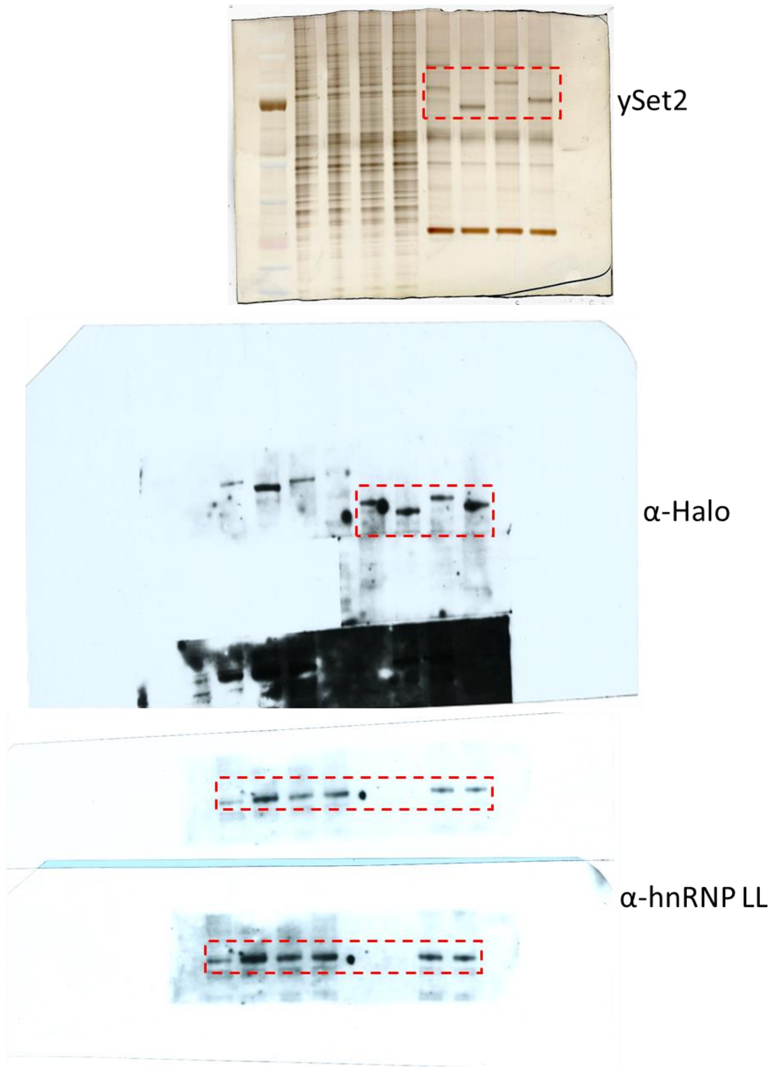

Figure 4

Figure 4b

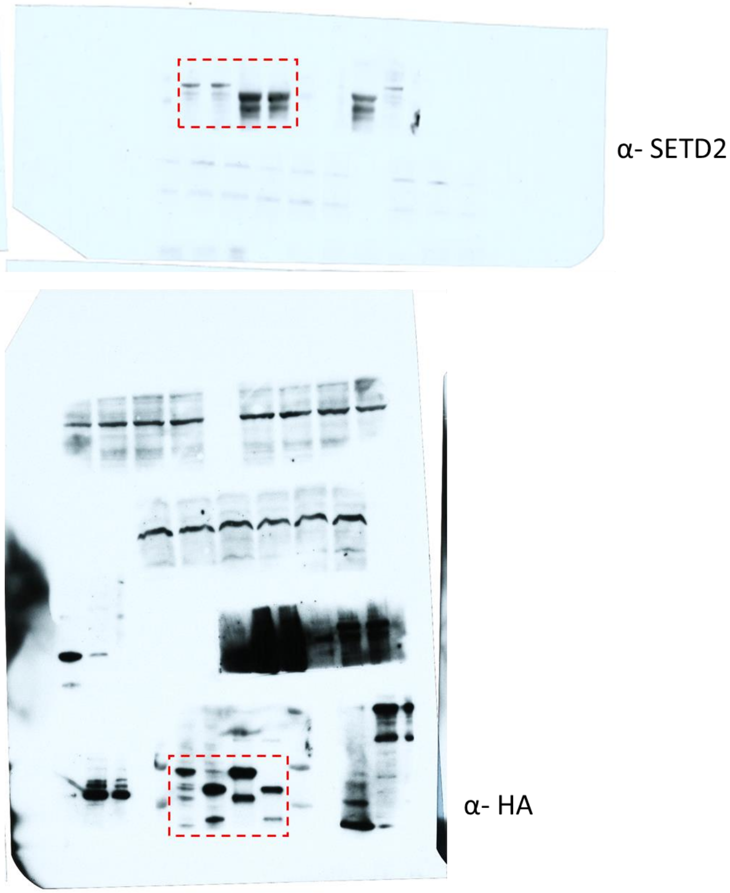

Figure 4c

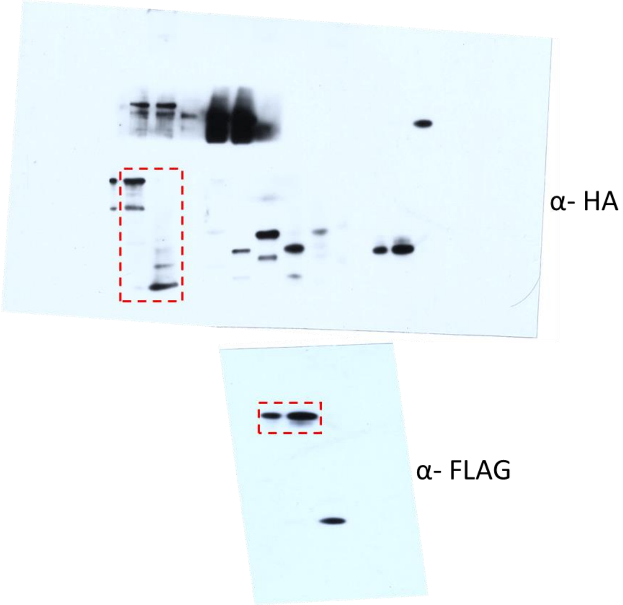

Figure 4e

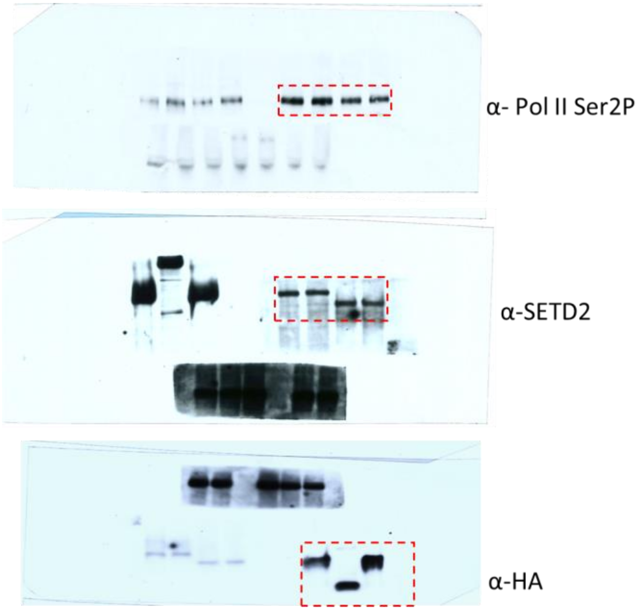

Figure 5

Figure 5b

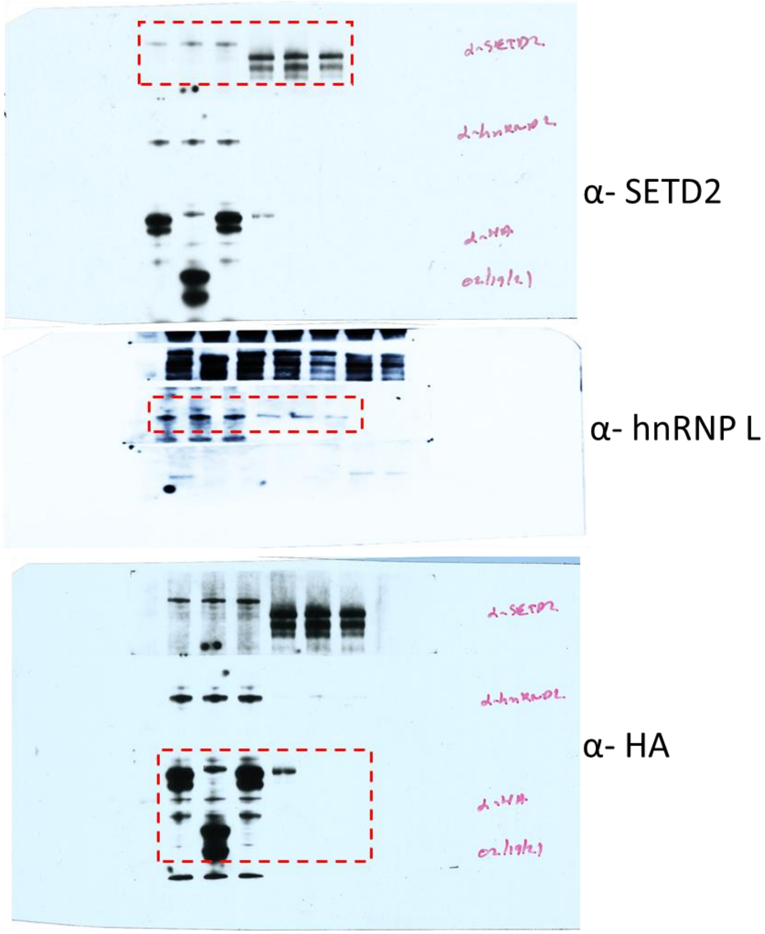

α- SETD2

α- hnRNP L

α- HA

Figure 5d

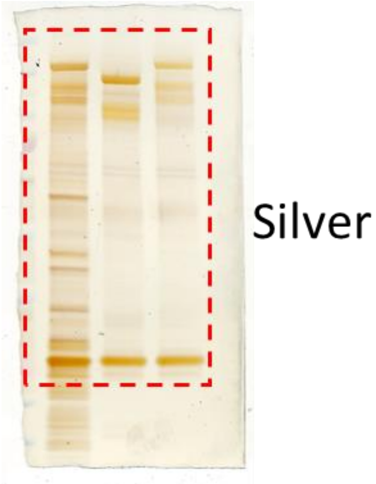

Silver

Figure 5e

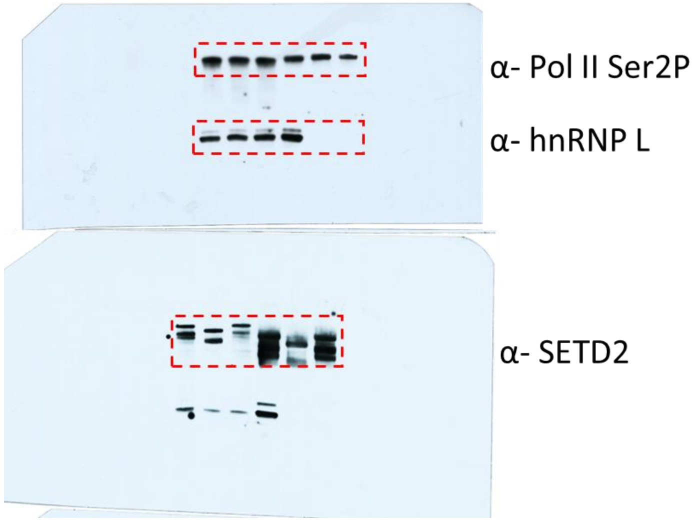

α- Pol II Ser2P

α- hnRNP L

α- SETD2

Figure 6

Figure 6h and l

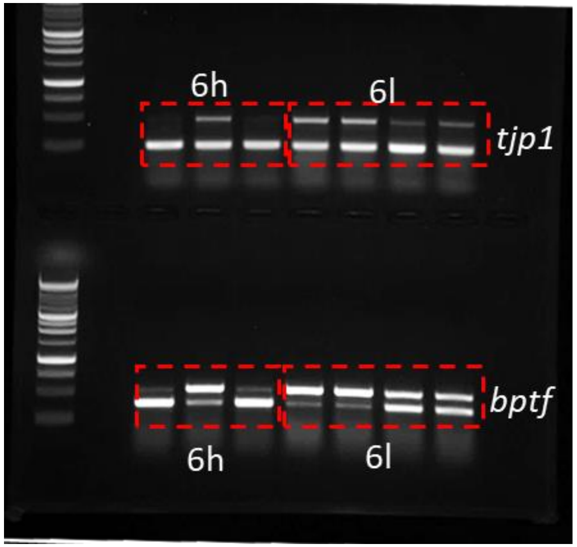

Supplementary Figure 5

Supplementary Figure 5a

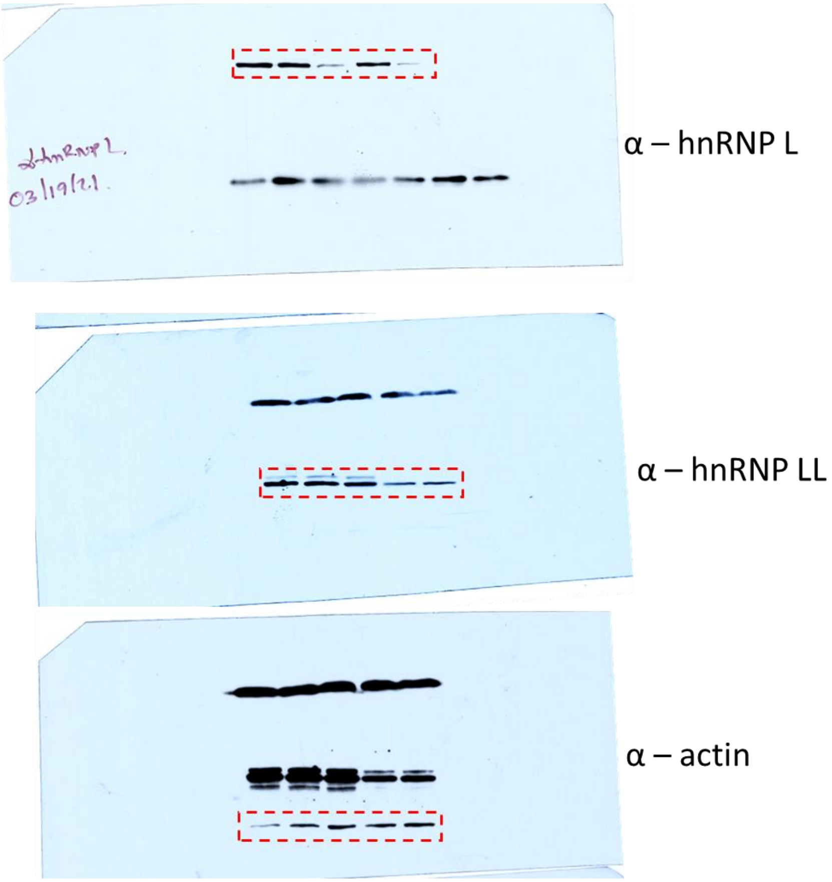

Supplementary Figure 6d

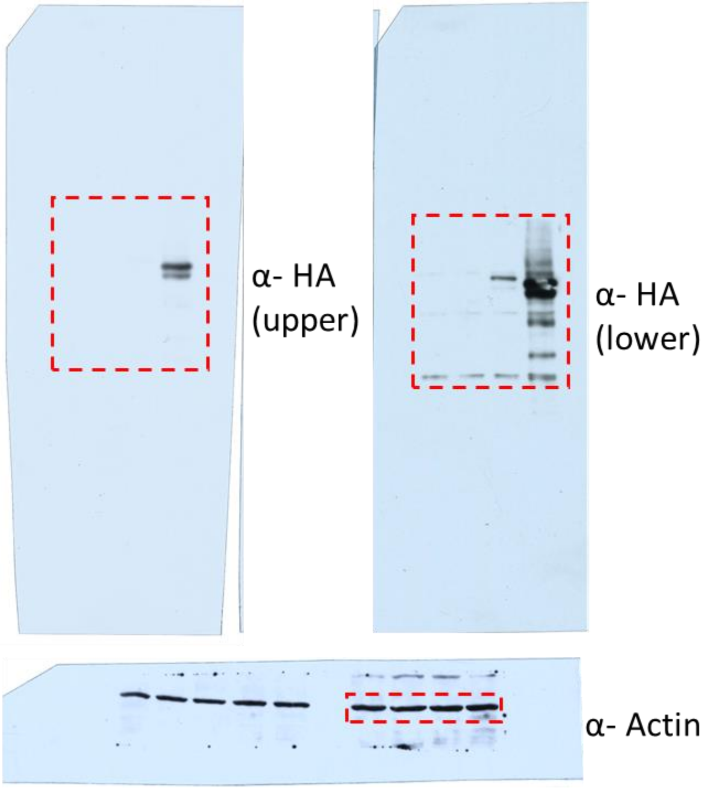

Supplement: Supplementary file 6 — Source Data [file 41467_2021_26799_MOESM6_ESM.pdf]
